# An Ensembled Anomaly Detector for Wafer Fault Detection

**DOI:** 10.3390/s21165465

**Published:** 2021-08-13

**Authors:** Giuseppe Furnari, Francesco Vattiato, Dario Allegra, Filippo Luigi Maria Milotta, Alessandro Orofino, Rosetta Rizzo, Rosaria Angela De Palo, Filippo Stanco

**Affiliations:** 1 Department of Mathematics and Computer Science, University of Catania, 95125 Catania, Italy; giuseppe.furnari@phd.unict.it (G.F.); francesco.vattiato@studium.unict.it (F.V.); allegra@unict.it (D.A.); fstanco@dmi.unict.it (F.S.); 2 STMicroelectronics, 95121 Catania, Italy; alessandro.orofino@st.com (A.O.); rosetta.rizzo@st.com (R.R.); rosaria.de-palo@st.com (R.A.D.P.)

**Keywords:** wafer fault detection, univariate and multivariate analyses, ensembled method

## Abstract

The production process of a wafer in the semiconductor industry consists of several phases such as a diffusion and associated defectivity test, parametric test, electrical wafer sort test, assembly and associated defectivity tests, final test, and burn-in. Among these, the fault detection phase is critical to maintain the low number and the impact of anomalies that eventually result in a yield loss. The understanding and discovery of the causes of yield detractors is a complex procedure of root-cause analysis. Many parameters are tracked for fault detection, including pressure, voltage, power, or valve status. In the majority of the cases, a fault is due to a combination of two or more parameters, whose values apparently stay within the designed and checked control limits. In this work, we propose an ensembled anomaly detector which combines together univariate and multivariate analyses of the fault detection tracked parameters. The ensemble is based on three proposed and compared balancing strategies. The experimental phase is conducted on two real datasets that have been gathered in the semiconductor industry and made publicly available. The experimental validation, also conducted to compare our proposal with other traditional anomaly detection techniques, is promising in detecting anomalies retaining high recall with a low number of false alarms.

## 1. Introduction

During the production process (PP) in the semiconductor industry, many machines and facilities with complex components can be used. Giving a general overview of the PP, we could list the following main phases: diffusion and associated defectivity test, parametric test, electrical wafer sort test, assembly and associated defectivity test, final test, and burn-in. A high number of involved phases implies a very likely chance of failure during the PP. For this reason, a meaningful number of tests are planned during the production, in order to grant the highest possible yield once the PP is completed. When a failure occurs, the yield decreases, sometimes even dramatically (an event called *crisis*). It would be desirable to understand which are the yield detractors and manage them with proper corrective actions. However, the equipment monitoring and diagnostic phases are challenging, as the PP can “evolve” through time due to factors such as ageing of the machines or changes of recipes. Among the many steps of the semiconductor manufacturing, Fault Detection (FD) plays a critical role to maintain a low number and the impact of the yield detractors [1]. During the FD, various sensors are leveraged to gather data about machines and products conditions. For instance, typical tracked parameters include: pressure, voltage, power, reference zero position, and pump status. For each one of these parameters, soft and hard control limits are defined in the product specifications. In the semiconductor environment, classic Statistical Process Control (SPC), methodologies such as Statistical Bin Limits (SBL) and Part Average Testing (PAT), required by automotive regulations [2,3], are used to assure the quality of each fabrication step [4,5]. Hence, any time one of the parameter has a value beyond the designed or statistical limits, then a warning is raised detecting the abnormal product. This represents the most common scenario for FD. However, sometimes a fault can occur even if all the tracked parameters are within the control limits. This is due to a limitation of the univariate analysis: in other words, a fault can be caused by a combination of two or more parameters. For handling this scenario, a multivariate analysis should be conducted instead. The scenario is even more complex, as the root cause of the fault could be unknown a priori [6]. Monitoring a PP through the FD tracked parameters, represented as multivariate time series (MTS) data, is a quite difficult task, both demanding for domain experts and critical for smart manufacturing [7]. Smart techniques for the analysis of FD parameters, often tracked among several machines, can be leveraged for triggering warnings or alarms based on intelligent decisions (predictive maintenance). This analysis based on artificial intelligence is part of the core elements of the fourth industrial revolution, indexed as industry 4.0. There is a growing interest in these topics, especially to increase the efficiency and security of the industrial environment [8,9].

In this work, we propose a new technique for anomaly detection in the semiconductor manufacturing context, in an unsupervised fashion (our data are actually labeled, but labels are used only for validation purposes). We are presenting an ensembled anomaly detector, employing both multivariate and univariate approaches. For balancing the outcomes of the two kinds of analysis, we also propose and compare three different voting systems. Our contribution can be summarized in:

- Definition of an ensemble method employing both univariate (ANOVA) and multivariate (OCSVM) approaches;
- Comparison of three balancing criteria for the ensembled method;
- Comparison of the proposed method with other classic anomaly detection techniques;
- Publication of two real semiconductor manufacturing scenario datasets.

### Related Work

Fault Detection, or anomaly detection, can be effectively addressed through multiple approaches, which differ from each other according to their key assumptions, application contexts, nature of input data, type of anomaly, and availability of annotated data. In recent decades, many methods have been proposed for anomaly detection. An anomaly detection method is proposed by An et al. [10] for using the reconstruction probability from the variational autoencoder. The reconstruction probability is a probabilistic measure that considers the variability of the distribution of variables. A model called the Deep Autoencoding Gaussian Mixture Model (DAGMM) is proposed by Zong et al. [11]. The DAGMM uses a deep autoencoder to generate a low-dimensional representation and reconstruction error for each input data point, which is further fed into a Gaussian Mixture Model (GMM). A hybrid model is proposed by Erfani et al. [12], where a deep belief network (DBN) is trained to extract generic underlying features, and a one-class SVM is trained from the features learned by the DBN. Additionally, in the manufacturing context, many methods are been proposed. Among them, some machine learning methods are benchmarked by Susto et al. [13]: the OsPCA [14], which is an anomaly detection method based on Principal Component Analysis (PCA), the OnlinePCA [15], which is another method based on PCA which aims to solve the OsPCA limitations, and Angle-Based Outlier Detection (ABOD) [16], where angles and feature spaces are leveraged for highlighting likely outliers. The ABOD’s key assumption is that normal samples will produce high variance angles while abnormal ones will be associated with low variance angles. The Local Outlier Factor (LOF) [17] considers local neighborhoods to compute an anomaly score, and the assumption key is that normal samples are a part of clusters, while an abnormal sample has a low-density neighborhood. These methods achieved satisfying performances [13]. More recently, approaches based on neural networks were proposed: a Stacked Autoencoder Learning for Anomaly Detection (SALAD) is presented by Vincent et al. [18]. The SALAD framework enables anomaly detection in real time by using a multidimensional time-frequency analysis of sensory data from fab tools. A modular neural network is presented by Hong et al. [19] and used with Dempster–Shafer (D–S) theory to perform fault detection and classification (FDC). Differently, a recurrent neural network based on long short-term memory [20] is used by Kim et al. [7] to predetect anomalies without any annotated data.

In our work, we present an ensembled anomaly detector based on One Class Support Vector Machine (OCSVM) [21] and Analysis of Variance (ANOVA) [22], leveraged to get advantages of both the multivariate (MVA) and univariate (UVA) analyses. To balance the contribution of both the UVA and MVA approaches in the anomaly detection, we define three different criteria. We also present two datasets from a real semiconductor industry context that we used to test our method. To the best of our knowledge, datasets of such kind are not yet publicly available. The SECOM Dataset [23] is an example of a similar available dataset, but it is really different from ours where there are temporally aggregated data of productions batch, different parameters (both in quantity and value ranges) and strategies for preprocessing missing values. Due to these differences, the SECOM dataset cannot be leveraged in our case. The lack of public datasets to be used as reference makes it difficult to compare new potential anomaly detection methods with the state-of-the-art ones.

The remainder of the paper is structured as follows: in Section 2, our datasets and the proposed anomaly detection method is described, while in Section 3, experiments and outcomes are discussed. Finally, in Section 4, the conclusion and possible future developments are given.

## 2. Materials and Methods

### 2.1. Dataset Overview

In this work, we used two different datasets concerning semiconductors industry. The datasets are publicly available at https://github.com/STMicroelectronics/ST-AWFD (accessed on 12 August 2021).

We refer to these datasets as *Dataset-1* (D1) and *Dataset-2* (D2). Both datasets contain time series made by a variable number of time samples and have 3 references features which are: Wafer-ID, Procedure-Step-ID and Timestamps. Moreover, two columns were added on both datasets: the Target column, which provides the label normal or abnormal for each wafer, and TestSet, which indicates if a time sample is part of the test set in our experiments, after the training-test sets’ splitting phase. The samples are grouped by a Wafer-ID, which represents the production lot and the single wafer (every lot contains 25 wafers). For instance, Wafer-ID *MAT0001.02* refers to the wafer *02* of the lot *MAT0001*. Then, the production process described is usually divided into steps. The number of steps is different between the two used datasets: 7 steps for D1 and 2 steps for D2. Two types of steps can also be defined: mandatory and optional ones. Finally, the timestamps denote the time samples of the time series. A preprocessing phase for timestamps was applied in order to convert the absolute reference time into a relative time representation. Since some steps can be optional, this means that the number of wafers with timestamps in each step can be variable. This behavior must be carefully considered when moving from an absolute to a relative representation of time. We will discuss the details of this time preprocessing phase in Section 2.5.

### 2.2. Dataset-1 (D1) Definition

*Dataset-1* (D1) contains an average of 200 time samples per wafer and a total of 5130 Wafer-IDs (giving a total of about 1 M time samples). During the production process, 44 measurements are collected in D1, resulting in a total number of about 230 K time series (for clarity, each time series contains an average of 200 time samples). There are five mandatory steps for D1 (namely, 2, 4, 5, 6, and 7), and there are two optional steps (−1 and −2). In this dataset, we have only two Wafer-IDs labeled as abnormal (we reiterate that labels are only used for model evaluation and not model training purposes).

### 2.3. Dataset-2 (D2) Definition

The *Dataset-2* (D2) contains an average of 100 time samples per wafer and a total of 2739 Wafer-IDs (giving a total of about 125 K time samples). During the production process, 22 measurements are collected in D2, resulting in a total number of about 25 K time series (for clarity, each time series contains an average of 100 time samples). In this case, there are only have two mandatory steps (and no optional steps). In D2, Wafer-IDs are labeled as abnormal through a temporal reference window.

### 2.4. Dataset Preprocessing

We have automatically removed measurements that were statistically meaningless. For instance, for each step we defined conditions on the minimum mandatory number of time samples and significant standard deviation of the time series (thus removing constant signals). Moreover, all the measurements have been standardized with a z-score scaler. All the preprocessing strategies we applied have been previously validated by a domain expert. After these preprocessing steps, we kept 15 measurements for 5104 wafers in D1 and 20 measurements for 1156 wafers in D2. Datasets were also anonymized for privacy purposes, as described in Appendix A.

### 2.5. Time Preprocessing

In our proposal, we needed to compare time series. This is a task that cannot be pursued by leveraging the absolute timestamps acquired during the production process. Hence, the absolute time reference, logged in UNIX format and milliseconds, was preprocessed in order to gain a relative representation. In this way, instead of considering when a time series begins and finishes, we just focused on its duration. To achieve this, we took the first time sample in the first step as the reference timestamp and calculated the difference of every time sample with the reference timestamp. The result obtained is the elapsed time from the reference timestamp to the sample timestamp (the duration). We also performed a min-max standardization of the duration, where the minimum value is 0 and the max is the last time sample duration (note that all time series have different absolute durations).

Since steps are distinguished as optional and mandatory; this difference must be handled in order to avoid wrong alignment after the time standardization. Indeed, if we do not distinguish these two kinds of steps, then optional steps may wrongly overlap the mandatory ones, introducing noise during the comparison of the time series.

To avoid this behavior, the min-max standardization was tuned using as reference min value the first time sample in the first mandatory step and as the reference max value the last time sample in the last mandatory step. After this preprocessing, we have a duration between 0 and 1 for the time samples in the mandatory steps, a duration lower than 0 for optional steps chronologically before the mandatory steps, and a duration greater than 1 for optional steps chronologically after the mandatory steps. Note that, in our datasets, optional steps are not allowed between two mandatory steps.

**Figure 1:**
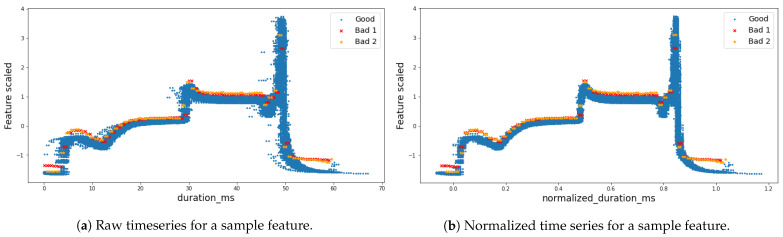
Time preprocessing: (**a**) the original raw time series and (**b**) the normalized representation, given a sample feature. Notice how, thanks to time preprocessing, the good samples are clustered together in a more compact baseline, while outliers are more separated from the baseline.

A comparison between raw and normalized time series for a sample feature is shown in Figure 1. Notice how in Figure 1b, thanks to time preprocessing, the good samples are clustered together in a more compact baseline, while outliers are more separated from the baseline.

### 2.6. Proposed Anomaly Detection Method

We began by analyzing the measurements one by one, in an *univariate* fashion. However, in a real manufacturing context this naive approach may not work, because the *excursions* (i.e., when an anomaly occurs) are often due to a combination of several metrics. Therefore, more than just a single measurement at a time should be considered during the anomaly detection, according to a *multivariate* paradigm. The proposed method will now be described in more detail.

Initially, we used a univariate analysis (UVA) approach for anomaly detection. The method consisted in building an One Class Support Vector Machine (OCSVM) [21] model for each step and for each measurement time series (i.e., in D1 we will have a number of models equal to 7 steps ×15 measurements). We counted the number of abnormal time samples detected by each model, and computed the abnormal rate as the ratio between the number of abnormal time samples and the total number of samples. The partial response of an OCSVM model is abnormal if given a step and a measurement with a negative rate greater than *N* times the standard deviation of the reference abnormal rate computed on the training set. Indeed, we adopted the Analysis of Variance (ANOVA) [22] as an outlier detection method. The OCSVM partial responses over the several steps and measurements are combined together to obtain a single OCSVM global response (i.e., a response for the whole process and not for a single step or measurement). Together with OCSVM, we also computed five aggregated statistics for each step and for each measurement time series: mean, standard deviation, range, min and max. The statistic partial responses were calculated through ANOVA, similarly to the OCSVM partial responses. When an aggregated statistic was detected as an outlier by ANOVA, we defined it as a triggered statistic. Therefore, an analyzed wafer could have a global number of triggered statistics between 0 and 5 for each step and for each measurement. Finally, OCSVM and statistics global responses are combined together, giving the final voting system.

To sum up, the naive UVA approach has these pros:

- Measurements with an anomalous behavior will likely have a high number of votes from ANOVA;
- The voting system is simple and the voters (i.e., OCSVM models and the 5 statistics) have all the same weights.

and these cons:

- Anomalies due to a combination of several measurements may not be detected by UVA OCSVM models;
- The number of the models increases with the number of parameters, which can rapidly increase the required computational power and time.

A second approach consisted of a fully multivariate analysis (MVA). To perform this analysis, we trained an OCSVM model for each step using all measurements at once. In this way, the model can learn how to recognize the anomalies due to combinations of several measurements. The OCSVM partial responses over the several steps were combined together to obtain a single OCSVM global response (i.e., a response for the whole process and not for a single step), similarly to UVA OCSVM global response. Therefore, MVA solves the limitations of the UVA method, but then loses the ability to detect the parameter(s) that likely caused the anomalies (especially using a nonlinear kernel function such as the common used Radial Basis Function RBF kernel). This will make the root-cause analysis harder, and it is not recommended in a real industrial scenario.

Therefore, looking for a trade-off between the mentioned UVA pros and cons, eventually we combined together the MVA and UVA approaches in an ensembled anomaly detector.

### 2.7. Balancing the Voting System of the Ensembler

In order to define the voting system of the ensembled anomaly detector, we need to balance the response of the MVA and UVA approaches, so we have defined three possible criteria:

- **EWC: equally weighted criterion**: 50% of the response is given by MVA and 50% is given by UVA;
- **MSC: MVA as a statistic criterion**: MVA response is weighted as one of the statistics computed in UVA;
- **SBC: score-based criterion**: weight MVA using the OCSVM score.

These criteria will be detailed in the following. However, despite the criterion, by summing the UVA and MVA responses for each step we can define an anomaly score for each wafer. By thresholding the anomaly score, we can eventually classify a wafer as normal or abnormal.

#### 2.7.1. EWC: Equally Weighted Criterion

Let *X* be the set of measurements, *S* the set of statistics, and *h* a function defined as h:S×X→N, where *h* takes *S* statistics and *X* measurements as input, and returns the *N* number of triggered statistics (as defined in Section 2.6). With $X_{w,t}$, we indicate the measurement set of the Wafer-ID *w* at the t−th step. The UVA score for a single wafer *w* at step *t* ($U_{w,t}$) is given by Equation (1), and it can be at most $|X_{w,t}|\times |S|$, where ∣·∣ denotes the number of elements in the set. The MVA score in the EWC ($M_{w,t}$) is given by Equation (2), where the MVA response (${\bar{M}}_{w,t}$) can be either 1 or 0 and it is weighted by a factor of $|X_{w,t}|\times |S|$. The total UVA score for a wafer *w* is the sum of UVA scores for each step (Equation (3), where *T* is the set of process steps). Similarly, the total MVA score for a wafer *w* is given as shown in Equation (4). Finally, the total score is given by adding together the total UVA and the total MVA scores, as shown in Equation (5).

(1) $$U_{w,t}=\sum_{x\in X_{w,t}}h\left(S,x\right)$$

(2) $$M_{w,t}={\bar{M}}_{w,t}\;\times |X_{w,t}|\times |S|$$

(3) $$UVA_{w}=\sum_{t\in T}U_{w,t}$$

(4) $$MVA_{w}=\sum_{t\in T}M_{w,t}$$

(5) $$Total_{w}=MVA_{w}+UVA_{w}$$

#### 2.7.2. MSC: MVA as a Statistic Criterion

Differently from EWC, in the MSC, the MVA response is weighted as a single statistic, so its response (either 1 or 0) is multiplied only by a factor ∣X∣, as shown in Equation (6), while the UVA score ($U_{w,t}$) and the total scores ($UVA_{w}$, $MVA_{w}$, $Total_{w}$) are calculated in the same way as previously illustrated

(6) $$M_{w,t}={\bar{M}}_{w,t}\;\times |X|$$

#### 2.7.3. SBC: Score-Based Criterion

The SBC is similar to the EWC, but in order to weight its response SBC leverages $s_{i}$, which is defined as the OCSVM score of the *i*-th time samples to weight their responses. For this purpose, we define two score accumulators. The first score accumulator is for the positive scores (PA: *Positive Accumulator*), as shown in Equation (7), while the second score accumulator is for the negative scores (NA: *Negative Accumulator*), as shown in Equation (7). The sum of PA and NA is 1. Through the accumulators, the distance from the boundary can be used as information to weight the MVA response. We used NA as a percentage of the MVA maximum possible value (Equation (8)), where ∣P∣ is the number of parameters. Finally, notice that when the negative accumulator is equal to 1, then SBC will behave as the EWC. The way in which the $U_{w,t}$, $UVA_{w}$, $MVA_{w}$ and the $Total_{w}$ are computed does not change.

(7) $$\begin{array}{cccccc} PA & =\frac{\sum_{i:s_{i}>=0}s_{i}}{\sum_{i}|s_{i}|} & \; & & NA & =\frac{\sum_{i:s_{i}<0}|s_{i}|}{\sum_{i}|s_{i}|} \end{array}$$

(8) $$M_{w,t}=NA\times {\bar{M}}_{w,t}\times |P|\times |S|$$

## 3. Results

**Figure 2:**
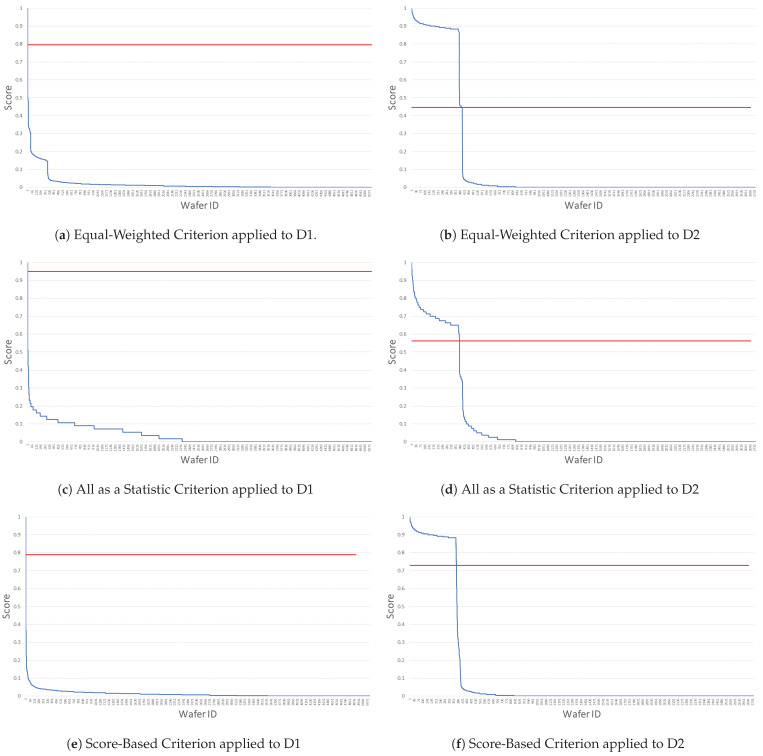
All balancing criteria applied for both the datasets. On the y axis we have the total score calculated by the criterion and on the x axis we have the Material-IDs (reported as increasing Wafer-IDs). In the first row is shown the results of equal-weighted criterion on D1 (**a**) and D2 (**b**), while (**c**,**d**) show results of All as a statistic criterion in D1 and D2, respectively. The results of the score-based criterion are shown in the final row for D1 (**e**) and D2 (**f**).

The three balancing criteria for the voting system of the ensembled anomaly detector (Section 2.7) have been tested on both D1 and D2 datasets. The total scores for each criterion are shown in Figure 2, where we have the total score (*y* axis) for each wafer (*x* axis); the first column represents the results of the EWC, MSC and SBC applied to D1, while the second column represents the result of the three criteria applied to D2. Notice that we have normalized the total score by a min-max scaler. The red line represents the threshold used to classify wafer as normal or abnormal depending on the total score.

For all the criteria, we empirically fixed a threshold for ANOVA equal to 3 times the standard deviation for the statistics responses in UVA and to 6 times the standard deviation for OCSVM responses (both in UVA and MVA). The outcomes for each criterion are reported in the following.

### 3.1. EWC: Equally Weighted Criterion

As can be seen in Figure 2a, in D1 there are few samples with high scores which belong to the abnormal class, while all the other ones belong to the normal class. In D2 (Figure 2b), similar patterns can be observed. The step edges which occur in the charts in Figure 2a,b are caused by the score of the MVA, which is far greater than the score of the UVA. The MVA score can be either 0 or 0.5, while the UVA score is a value in the range [0,0.5]. Thus, we need to change the contribution of the MVA score, which led us to using the MVA as a statistic criterion.

### 3.2. MSC: MVA as a Statistic Criterion

With this criterion (Figure 2c,d), we smoothed the steps of the first dataset. However, in both datasets, MSC identifies more abnormal wafers than the equal-weighted criterion. However, in this case, we have the opposite problem: the MVA approach is not good enough for the outlier detection task.

### 3.3. SBC: Score-Based Criterion

As showed in Figure 2e,f, the use of the negative accumulator led to better results. This criterion produces a clearer division between the normal and the abnormal classes. This makes it easier to find a threshold for both datasets.

### 3.4. Experimental Results for the Balancing Criteria

In order to distinguish the bad materials from the good ones, for each criterion EWC, MSC and SBC, we found a score threshold using a flex point search algorithm. Given the defined threshold, we can evaluate each criterion. Important information is given by the confusion matrix in Table 1, where it is clear that the SBC manages false alarms and bad misses better than the EWC and MSC. Hence, we have two true negatives and one false alarm in D1 using the EWC (in D1 we have just two actual bad wafers), while using the MSC we detected just one true negative and missed the second; therefore, SBC exhibits the better performance with both the two true negatives detected and 0 false alarms. A similar behavior is shown in D2, where we have 37 false negatives (false alarms) with the EWC, decreasing to 14 with the MSC and to 5 with the SBC criterion.

### 3.5. Experimental Results Comparing Other Methods

Our proposed method has been compared with the following anomaly detection techniques [24]:

- One Class Support Vector Machine (OCSVM) [21];
- Copula-Based Outlier Detection (COPOD) [25];
- Fast Angle-Based Outlier Detection (FABOD) [16];
- Isolation Forest (IF) [26].

Each model has been trained on the same training set and for each Wafer-ID we computed the negative rate, similarly as described for classifying outliers in Section 2.6. If the negative rate is greater than a given threshold, the Wafer-ID is classified as abnormal. In our test, we set the threshold to 0.5 (50% of negative samples). For each method, we performed several experiments, varying hyperparameters through a grid search approach. The best results represented as confusion matrices are summarized in Table 2. In dataset D1, all the compared methods were able to correctly classify all the true positives, returning 0 false negatives. However, only the proposed method and FABOD were able to catch the two true negatives (the anomalies). Instead, in Dataset D2, OCSVM showed the best performances, correctly classifying all the true negatives and mistakenly classifying only one false negative. FABOD had a higher number of false negatives when compared to our proposal. COPOD also classified only one false negative, but mistakenly classified a very high number of false positives. Hence, for D1, our proposal and FABOD were the best methods for outlier detection, while for D2 OCSVM was the best method. However, our proposal was very close to the performances of OCSVM, with just a little gap of four false negatives. This behavior can be justified by the trade-off between the MVA and UVA analyses, as OCSVM is based only on the MVA. In D2, the UVA analysis introduced a bit of noise, resulting in a little gap of false negatives when MVA and UVA were balanced together in our proposed ensemble method.

## 4. Conclusions

In this work, we presented the following major contributions:

- Definition of an ensemble method employing both univariate (ANOVA) and multivariate (OCSVM) approaches;
- Comparison of three balancing criteria for the ensembled method;
- Comparison of the proposed method with other classic anomaly detection techniques;
- Publication of two real semiconductor manufacturing scenario datasets.

In detail, we have presented an ensemble between Univariate (UVA) and Multivariate (MVA) approaches to handle anomaly detection in a real manufacturing context. To combine the UVA and MVA responses, we have proposed three different balancing criteria: Equally Weighted Criterion (EWC), MVA as a Statistic Criterion (MSC) and Score-Based Criterion (SBC), used to balance the weights of the defined voting system. The results achieved in our datasets show that SBC is the best criterion to balance the UVA and MVA contributions and to obtain the best performances intended as the lower number of false alarms and the bigger number of true negatives (abnormal wafers) detected. Our proposed method has been compared with several anomaly detection techniques [24]: One Class Support Vector Machine (OCSVM), Copula-Based Outlier Detection (COPOD), Fast Angle-Based Outlier Detection (FABOD), and Isolation Forest (IF). Through this benchmark, the proposed method proved to have top performances for both the employed datasets. Finally, the release of new public semiconductor manufacturing datasets (https://github.com/STMicroelectronics/ST-AWFD, accessed on 12 August 2021) can be a baseline for comparing other anomaly detection methods and also for other researchers for possible future investigations. Other promising future research may focus on testing deep learning oriented anomaly detection methods, or may aim to expose relevant correlations among process parameters, in order to highlight meaningful root causes, resulting in more complex and clever anomaly detection procedures.

## Figures and Tables

**Table 1:** Confusion matrix for all balancing criteria and both datasets. For each confusion matrix we have true negatives (true abnormal Wafer-ID detected) and false negative (normal wafer classified as abnormal) on the first row, while on the second row we have the false positive (abnormal wafer classified as normal) and true positive (true normal wafer).

			D1		D2	
		Actual	Abnormal	Normal	Abnormal	Normal
	Predicted					
EWC	Abnormal		2	1	367	37
	Normal		0	5101	0	752
MSC	Abnormal		1	0	367	14
	Normal		1	5102	0	775
SBC	Abnormal		2	0	367	5
	Normal		0	5102	0	784

**Table 2:** Comparison for Datasets D1 and D2 of our proposed method (PROPOSED) with several classic anomaly detection methods: OCSVM, COPOD, FABOD, and IF. The comparison is presented as a benchmark of confusion matrices, reporting True Negative (TN), False Positive (FP), True Positive (TP), and False Negative (FN).

DATASET	METHOD	TN	FP	TP	FN
D1	PROPOSED	2	0	5102	0
	OCSVM [21]	0	2	5102	0
	COPOD [25]	0	2	5102	0
	FABOD [16]	2	0	5102	0
	IF [26]	0	2	5102	0
D2	PROPOSED	367	0	784	5
	OCSVM [21]	367	0	788	1
	COPOD [25]	189	178	788	1
	FABOD [16]	367	0	777	12
	IF [26]	367	0	731	58

**Figure A1:**
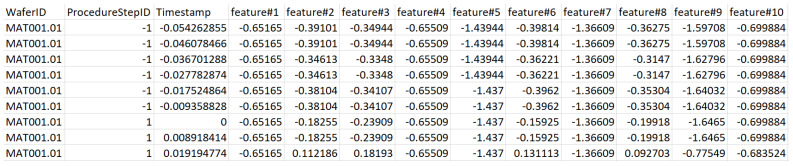
Dataset masking and anonymization: some sample rows.

## Data Availability

Refer to the dataset availability note we added in the last page.

## Abbreviations

The following abbreviations are used in this manuscript:

ABOD	Angle Based Outlier Detection
ANOVA	ANalysis Of VAriance
COPOD	Copula-Based Outlier Detection
D1	Dataset-1
D2	Dataset-2
DAGMM	Deep Autoencoding Gaussian Mixture Model
DBN	Deep Belief Networks
EWC	Equally Weighted Criterion
FABOD	Fast Angle Based Outlier Detection
FD	Fault Detection
FDC	Fault Detection and Classification
GMM	Gaussian Mixture Model
IF	Isolation Forest
LD	Linear Dichroism
LOF	Local Outlier Factor
$M_{w,t}$	The MVA score for a single wafer *w* at the step *t*
${\bar{M}}_{w,t}$	The MVA response for a single wafer *w* at the step *t*
MSC	MVA as a Statistic Criterion
MTS	Multivariate Time Series
MVA	Multivariate Approach
NA	Negative Accumulator
OCSVM	One Class Support Vector Machine
PA	Positive Accumulator
PAT	Part Average Testing
PCA	Principal Component Analysis
PP	Production Process
SALAD	Stacked Autoencoder Learning for Anomaly Detection
SBC	Score Based Criterion
SBL	Statistical Bin Limits
SPC	Statistical Process Control
$U_{w,t}$	The UVA score for a single wafer *w* at the step *t*
UVA	Univariate Approach

## Appendix A. Dataset Masking and Anonymization

Even if the two datasets presented in the paper in Section 2.1 were acquired in real use-case scenarios, we cannot disclose confidential information. Hence, we carefully masked and anonymized any sensible data. For the sake of clarity, an extraction of some sample rows from of one of the published datasets is shown in Figure A1.
